# Kocuria rosea Bacteremia in a Sickle Cell Patient: A Case Report

**DOI:** 10.7759/cureus.28870

**Published:** 2022-09-06

**Authors:** Brian G Nudelman, Taylor Ouellette, Kimberly Q Nguyen, William H Schmaus, Rajiv R Chokshi

**Affiliations:** 1 Internal Medicine, Nova Southeastern University Dr. Kiran C. Patel College of Osteopathic Medicine, Fort Lauderdale, USA; 2 General Surgery, Nova Southeastern University Dr. Kiran C. Patel College of Osteopathic Medicine, Fort Lauderdale, USA; 3 Internal Medicine, Broward Health Medical Center, Fort Lauderdale, USA

**Keywords:** gram positive bacteremia, immunocompromised patient, staphylococcus sp, hospital aquired, nosocomial infection, kocuria rosea, gram positive bacteria, sickle cell disease (scd), hospital epidemiology, catheter related blood stream infections

## Abstract

Central line-associated bloodstream infections (CLABSI) are a significant risk factor for poor patient outcomes. It is important to correctly diagnose and treat these infections to ensure the best chance of recovery. *Kocuria rosea* is a novel bacteria that is native to the human flora and has been on the rise as a culprit in recent nosocomial infections. However, due to its characteristics, it is often misclassified by commonly used hospital tests. We present a case of a 55-year-old female with sickle cell disease who developed a *K. rosea* infection during her hospital course and was effectively diagnosed and treated. This case report aims to bring awareness to this unusual bacteria as a possible cause of inpatient infection and CLABSI. Further research should be conducted to determine the incidence of this bacteria and the best testing to be done for its proper recognition.

## Introduction

Infections due to the genus Kocuria are extremely rare. The Kocuria genus is composed of Gram-positive cocci and belongs to the Micrococcaceae family, the Actinomycetales order, and the Actinobacteria class. Kocuria rosea is one of 19 Kocuria species identified [1]. They are aerobic, coagulase-negative, and catalase-positive bacteria. K. rosea grows in pairs, clusters, or tetrads on simple media and can be smooth or rough in appearance. The colors range from orange to red and pink. Growth occurs at an optimal temperature of 25-37 °C [2]. There is speculation as to why there are so few reports of infection with this organism. It is partly due to being ignored as a laboratory contaminant and also due to the phenotype-based identification assays that are used and the incorrect diagnosis of staphylococcal pathologies [1]. What sets micrococci apart from staphylococci are Kocuria’s sensitivity to bacitracin and lysozyme and its resistance to nitrofurantoin and lysostaphin [1]. Although these organisms are mostly environmental, inhabiting ecological niches and the soil, they are also found on human skin and oropharyngeal mucosa [2]. The few cases in the literature to date include immunocompromised individuals, patients on peritoneal dialysis, and catheter-related bacteremia [3,4]. However, further literature regarding this bacteria, its clinical presentation, or the risk factors is lacking.

## Case presentation

We present a case of a 55-year-old African American female who presented to the emergency department with a fever and a sickle cell pain crisis, which consisted of pain in the arms, legs, and chest. Her past medical history includes sickle cell disease, hypertension, anemia, a brain aneurysm five years ago, and a recent oral gingival abscess infection.

At the first encounter, she was febrile at 101.4 °F and her respiratory rate was 18 breaths per minute with an oxygen saturation of 99% on room air. Her heart rate was 99 beats per minute with a blood pressure of 107/65 mmHg. An X-ray done in the emergency department was unremarkable and showed mild cardiomegaly. Her EKG was negative. She was admitted to telemetry for further monitoring and care. The patient was continued on hydroxyurea 1500 mg, folic acid 1 mg, and a heating pad. She was also placed on a pain regimen of oxycodone 10 mg every 12 hours and gabapentin 300 mg every eight hours. Blood cultures were positive for K. rosea 2/2. The patient was transfused with 1 unit of packed red blood cells due to hemoglobin below her baseline of 6. The patient was placed on deferoxamine 1 g by hematology. Infectious disease placed the patient on IV vancomycin from February 19 to February 24 and IV Meropenem from February 19 to February 23. The patient was also started on levofloxacin 750 mg P.O. daily and minocycline 100 mg P.O. BID for seven days from February 19 to February 26. Vascular surgery was consulted for port removal due to positive blood cultures. The port removal was successful with no complications. A Penrose drain was placed and removed with no complications or infections. The patient had positive extended-spectrum beta-lactamase (ESBL) bacteriuria but was asymptomatic and was discharged with oral antibiotics.

## Discussion

This presentation demonstrates an interesting case of *K. rosea* bacteremia in a sickle cell patient. This bacteria is a natural flora of the human integumentary system that can become a cause of hospital-acquired infection or central line-associated bloodstream infections (CLABSI) in immunocompromised patients [5]. The usual culprits of device-related septicemia include Gram-negative bacilli, *Staphylococcus aureus*, Coagulase-negative staphylococci, and *Candida albicans* [2]. However, with Kocuria species emerging as catheter-infecting organisms, it is important that they do not continue to be misidentified by phenotypic tests or dismissed as laboratory contaminants. The table below demonstrates just how similar Kocuria and Staphylococci are but that there are distinct differences that can be used to set them apart [2,6]. To avoid misidentification, healthcare systems can implement bacitracin or lysozyme and nitrofurantoin or lysostaphin tests into sensitivity assays [2].

Although the majority of infections caused by *K. rosea* are in immunocompromised patients, complications from this bacteria can even be seen in relatively healthy patients. A case report in 2013 discussed a case of descending necrotizing mediastinitis in a 58-year-old woman with a history of only significant gout and hypertension [5]. Another report from 2013 analyzed a case of rheumatic mitral valve endocarditis caused by *K. rosea* [7]. Both cases demonstrated that this bacteria can cause serious issues in patients with no significant immunosuppression. This also stresses the importance of avoiding a delay in the recognition of the bacteria by culture. Micrococci are a common cause of sample contamination during culture; due to Kocuria’s similarity to this genus, it is often disregarded as an insignificant finding [8]. This simple fact can lead to the progression of simple infections to more critical conditions.

**Table 1:** Comparison of Kocuria vs. Staphylococci

Characteristics	Kocuria	Staphylococci
Gram stain	Gram-positive	Gram-positive
Shape	Spherical	Spherical
Arrangement	Tetrads, pairs, packets, clusters, or single cells	Pairs and grape-like clusters
Oxygen requirement	Aerobic	Facultatively anaerobic
Spores	Non-spore forming	Non-spore forming
Motility	Non-motile	Non-motile
Catalase test	Catalase-positive	Catalase-positive (can be negative)
Nitrofurantoin sensitivity	Resistant	Sensitive (may express resistance)
Lysostaphin sensitivity	Resistant	Sensitive
Bacitracin sensitivity	Sensitive	Resistant
Lysozyme sensitivity	Sensitive	Resistant

## Conclusions

In summary, our report illustrates a rare case of *K. rosea* bacteremia in a patient with known sickle cell disease. In phenotypic assays, *K. rosea* is often misidentified as a Staphylococcus species. Using drugs that routinely treat Staph infections may not work on *K. rosea* due to its resistance to nitrofurantoin and lysostaphin. Lack of adequate antibiotic treatment in the setting of a catheter-related infection can frequently cause progression to bacteremia. It is vital to identify the correct bacteria to prevent these complications, not only in immunocompromised individuals such as our patients but also in relatively healthy individuals. Current data on *K. rosea* are sparse, but this report helps to emphasize the need for more identification-related and epidemiologic research studies.
